# Compromised endothelial progenitor cell exosomal communication with endothelial cells in hypertension ischemia conditions

**DOI:** 10.3389/fstro.2022.1015463

**Published:** 2022-10-13

**Authors:** Shuzhen Chen, Venkata Polaki, Ji C. Bihl, Jinju Wang

**Affiliations:** 1 Department of Biomedical Sciences, Joan C. Edwards School of Medicine, Marshall University, Huntington, WV, United States,; 2 Department of Pharmacology and Toxicology, Boonshoft School of Medicine, Wright State University, Dayton, OH, United States

**Keywords:** exosomal communication, endothelial progenitor cells, endothelial cells, neurons, hypertension, ischemia stroke

## Abstract

We have previously demonstrated that endothelial progenitor cell (EPC) derived exosomes (EPC-EXs) can protect endothelial cells (ECs) against hypoxia injury. Given that EX function varies upon the cellular status and EPC function is declined in hypertension, we speculate the function of EPC-EXs is altered in hypertension-ischemia conditions. Here, we studied the EPC-EX mediated communications of EPCs with ECs in hypertension-ischemia conditions. EPC-EXs were prepared from the bone marrow EPCs of wild-type (WT) and hypertensive renin transgene (R+) mice (WT-EPC-EXs and R-EPC-EXs, respectively). To mimic hypertension-ischemia injury, ECs were challenged with angiotensin II (Ang II; 10^–6^ M) plus hypoxia (1% O_2_ for 6 h) and reoxygenation (21% O_2_ for 24 h). To determine the function of EPC-EXs, ECs were co-cultured with EXs during the reoxygenation period. EX uptake effciency, EC viability, and angiogenic function were assessed. We found that: (1) The incorporation effciency of R-EPC-EXs by ECs was significantly decreased compared to the WT-EPC-EXs. (2) Ang II plus hypoxia reoxygenation-injured ECs displayed decreased cell viability, increased cell apoptosis, and compromised angiogenic ability, which were alleviated by R-EPC-EXs. (3) WT-EPC-EXs elicited better effects than R-EPC-EXs on protecting ECs from hypertension plus hypoxia injury. In conclusion, our data have demonstrated that EPC-EXs mediated communication of EPCs and ECs is compromised in hypertension-ischemia conditions, suggesting that impairment of EPC exosomal communication might contribute to the exaggerated cerebral ischemia injury in hypertension-associated ischemic stroke.

## Introduction

According to the Centers for Disease Control and Prevention (CDC) report, ~795,000 stroke events occur annually in the United States. As the most prevalent risk factor for ischemic stroke, hypertension affects nearly 1/3 of the US population and presents in up to 84% of acute stroke patients (Feigin et al., 2016). Preclinical evidence shows that the ischemic brain damage is significantly enlarged in mouse genetic models of hypertension (Chen et al., 2013; Cipolla et al., 2018). The underlying mechanism might be related to the activation of the renin-angiotensin system, which contributes to endothelial dysfunction and blood-brain barrier leakage (Cipolla et al., 2018; Setiadi et al., 2018). Therefore, protecting endothelial cells (ECs) against acute ischemic injury might be an important strategy in the clinical management of ischemic stroke.

Endothelial progenitor cells (EPCs), a new subset of stem cells in the circulation and bone marrow, are gaining intense attention in stroke research. EPCs are involved in maintaining appropriate endothelial function (Yoder, 2012), maintaining the integrity and function of the brain vessels (Guo et al., 2017). In response to vascular injury, EPCs mobilize from the bone marrow to home into the site of ischemia and repair the vascular endothelium (Condon et al., 2004). In the ischemic tissue, EPCs could differentiate and mature into ECs and become part of the new vasculature (Ingram et al., 2005; Hu et al., 2010), repairing endothelial damage through the secretion of growth factors such as vascular endothelial growth factor and stromal cell-derived factor 1 to recruit more EPCs (Evans et al., 2021). EPCs can also induce angiogenesis or vasculogenesis to protect the neurovascular unit in the brain (He et al., 2011). We have previously demonstrated that infusion of EPCs could alleviate cerebral ischemia injury (Chen et al., 2013). Unfortunately, the number and repair capacity of EPCs are substantially declined in hypertension conditions (Oliveras et al., 2008; Luo et al., 2016), which might contribute to the poor prognosis in hypertension-associated ischemic stroke.

Increasing evidence shows that stem/progenitor cell-derived extracellular vesicles have great therapeutical potential in regenerative medicine (Mattingly et al., 2021). Exosomes (EXs), a type of extracellular vesicle, contain a variety of biological factors. A large body of evidence indicates the potentiality of EXs as a novel mechanism for the cross-talks of cell-to-organ/tissue (Deregibus et al., 2007; Whitham et al., 2018). Progenitor/stem cell-derived EXs such as EPC-EXs and mesenchymal stem cell-derived EXs have great therapeutical potential in cardiovascular diseases. Liu and colleagues have demonstrated that EPC-EXs could inhibit neointimal hyperplasia by promoting re-reendothelialization and suppressing the proliferation and migration of smooth muscle cells (Kong et al., 2018). We have previously shown that EPC-EXs protect ECs against hypoxia injury *in vitro* and *in vivo* (Wang et al., 2013, 2020a).

Of note, EX functions vary upon cellular origin and status (Yanez-Mo et al., 2015; Doyle and Wang, 2019; Zhang et al., 2019). EXs from visceral adipose tissue of obese mice could exhibit pro-inflammation effects, which promote the progression of atherosclerosis in hyperlipidemic apolipoprotein E-deficientmice (Xie et al., 2018). The circulating endothelial extracellular vesicles are implicated in diabetes-associated cerebral ischemic damage (Chen et al., 2011). We have demonstrated that extracellular vesicles released from starved EPCs exhibited opposite anti-apoptosis and anti-oxidative effects in ECs to those released in inflammation condition (Wang et al., 2013), suggesting that EPC-EXs-mediated communication is functional and modulable by factors that affect the status of their parent cells. Clinical studies revealed that the function of EPCs declined in patients with hypertension (Oliveras et al., 2008; Yu et al., 2019); whether the function of EPC-EXs is altered in hypertension conditions is unknown.

Given the importance of EPC-EC communication for vascular endothelial repair in ischemic stroke, we investigated the exosomal communication between EPCs and brain ECs in hypertension conditions in this study.

## Methods

### Animals

The renin hypertensive transgene (R+) mice and wild-type (WT) controlmice (male and female, 15–16-week-old) were used in this study. All mice were maintained in a 22°C room with a 12 h light/dark cycle and fed with standard chow and drinking water *ad libitum*. Body weights were recorded weekly. All experimental procedures were approved by the Application for the Care and Use of Laboratory Animals at Marshall University (IACUC) and were in accordance with the Guide for the Care and Use of Laboratory Animals issued by the National Institutes of Health.

### Isolation of EPCs from bone marrow

EPCs were cultured from the bone marrow (BM) of R+ and WT mice, as we previously reported (Chen et al., 2013). In brief, femurs and tibias were collected, and the bone marrow was flushed out using a 1mL syringe with 1mL PBS. Then the bone marrow was gently layered over 2ml of gradient medium (Hisopaqu-1083, Cat # 10831; Sigma-Aldrich, Inc.) for centrifugation at 800 g for 30min at 4°C. The mononuclear cells in the interface layer were collected into a new 15ml tube and washed with 1ml PBS by centrifugation at 400 g for 5min at 4°C. The cell pellets were resuspended with EC basal medium−2 (EBM-2) supplemented with EPC growth factors (Cat # CC-3162; Lonza) and seeded into tissue culture T25 flasks. After 2 days in culture, non-adherent cells were removed by washing with PBS. After that, the culture medium was changed every 2 days.

### Preparation and characterization analysis of EPC-EXs

After 7 days of culture of EPCs, the cell culture medium was changed to serum-free culture medium supplemented with growth factors to stimulate EX release for 2 days. To harvest EXs, the culture medium was collected and centrifuged at 300 g for 15min, followed by 2,000 g for 30min to remove dead cells and cell debris. Then the cell-free medium was centrifuged at 20,000 g for 70min to remove microvesicles, and then ultracentrifuged at 170,000 g for 90min to pellet EXs. The harvested EXs were resuspended with 20nm filtered (Whatman, Pittsburgh, PA) phosphate-buffered saline (PBS) and aliquoted for Nanosight tracking analysis (NTA), western blot, and transmission electron microscopy, as we previously reported (Wang et al., 2013). Some EXs resuspended with the culture medium of ECs for co-culture experiments.

### Induction of hypertension-hypoxia cell injury model

A mouse brain microvascular ECs (Cell Biologics, Inc; Cat # C57–6023) were used in this study. The ECs were maintained and expanded according to the manufacturer’s instructions. To mimic the hypertension-ischemia injury *in vitro*, ECs were challenged by Ang II (10^−6^ M) (Gu et al., 2014) plus hypoxia/reoxygenation (H/R; 1% O_2_ for 6 h followed by 21% O_2_ for 24 h) as we previously reported (Wang et al., 2013). The cell models were analyzed by MTT, apoptosis and tube formation assays.

### Co-culture of EPC-EXs with hypertension-hypoxia challenged ECs

To determine the function of EPC-EXs, EC injury model cells were randomly divided into three treatment groups (*n* = 6/subgroup/measurement): vehicle (culture medium only), WT-EPC-EXs, and R-EPC-EXs. The concertation of EPC-EXs used for the co-culture study was 4 × 10^7^ EXs/ml (Wang et al., 2020b). After 24 h co-culture, cellular survival ability, including methyl thiazolyl tetrazolium (MTT), apoptosis rate, and function capability (tube formation ability), were analyzed.

### MTT and apoptosis assays of ECs

After 24 h co-culture with WT-EPC-EXs or R-EPC-EXs, the cell survival ability was evaluated bymethyl thiazolyl tetrazolium (MTT, Cat # V13154; Invitrogen) method (Wang et al., 2013) and Annexin V/PI staining kit (Cat # BD556547; BD Bioscience) according to the manufacturer’s instruction.

### Tube formation ability analysis of ECs

The *in vitro* angiogenesis ability of ECs in different experimental groups was determined using a tube formation assay kit (Cat # ECM625; FisherScientific), as we previously described in Chen et al. (2014). Briefly, the ECMatrix solution was thawed on ice overnight, mixed with 10x ECMatrix diluent buffer, and placed in a 96-well tissue culture plate at 37°C for 1 h. Then the cells were replated (1 × 10^4^ cells/well), seeded and incubated for 24 h at 37°C. Tube formation was evaluated with an inverted lightmicroscope and defined as a tube structure exhibiting a length 4 times its width. Five independent fields were assessed for each well, and the average number of tubes per field (magnification, 200x) was determined.

### Uptake efficiency analysis of EPC-EXs with ECs

To determine the uptake efficiency of EPC-EXs by ECs and neurons, the EXs were labeled with PKH 67 (2 uM; Cat # PKH67GL; MilliporeSigma) according to the manufacturer’s instructions. In brief, EX pellets were resuspended with 2 *μ*M PKH67 dye in 1ml PBS for 5min at RT, then 1ml FBS was added to stop staining, followed by ultracentrifugation. The labeled EXs were resuspended with EC culture medium (4 × 10^7^ EXs/ml) for co-culture experiments.

### Western blot analysis

Standard western blot protocol was used for measuring the protein levels of exosomal specific markers: CD63 (1:500, Cat # 216130; Abcam), Tsg 101 (1:1000, Cat # ab30871; Abcam); EPC specific markers: CD34 (1:1000, Cat # ab81289; Abcam), VEGFR2 (1:1000, Cat # 55B11; Cell Signaling Technology); Cleaved cas-3 (1:200, Millipore), and β-actin (1:4000, Sigma).

### Statistical analysis

Data are expressed as the mean ± SEM. Multiple comparisons were analyzed by one- or two-way ANOVA followed by the Tukey *post hoc* test. GraphPad Prism 9 was used for analyzing the data. For all measurements, *p* < 0.05 was considered statistically significant.

## Results

### The incorporation efficiency of EPC-EXs from hypertensive R+ mice by ECs is decreased

**FIGURE 1:**
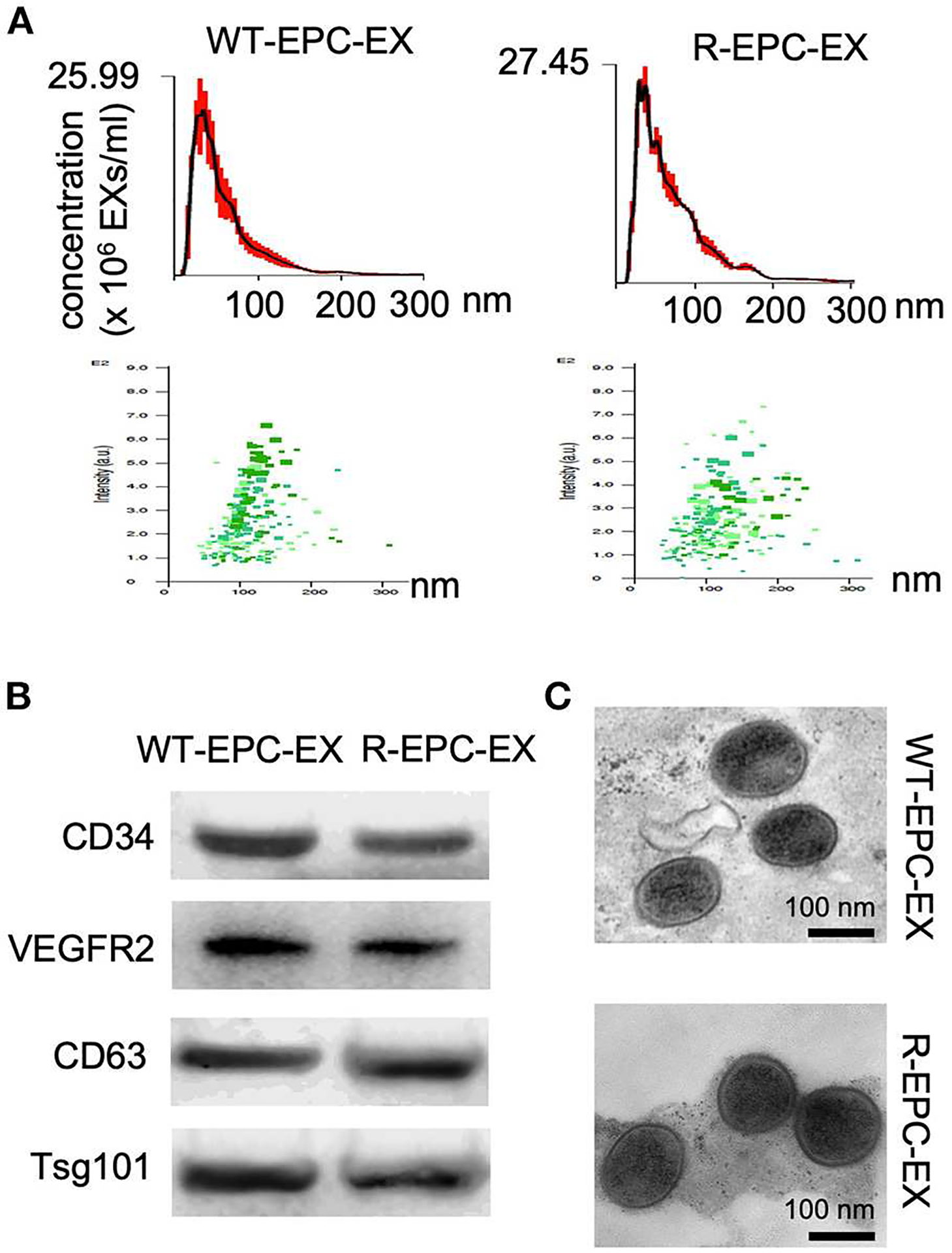
Characterization of EPC-EXs derived from WT and R+ mice. **(A)** size distribution and concentration of EPC-EXs; **(B)** Exosomal and EPC specific marker expression in EPC-EXs as determined by western blot analysis; **(C)** representative of EX structure as assessed by TEM. Scale bar: 100 nm.

First, we used multiple approaches to characterize the EXs released by the bone marrow EPCs of WT and R+ mice (denoted as WT-EPC-EXs, R-EPC-EXs). Our results showed that both had WT-EPC-EXs and R-EPC-EXs with an average size < 150nm, positively expressed EPC (CD34 & VEGRF2) & exosomal specific markers (CD63 & Tsg101) and were in cup-shape (Figure 1).

**FIGURE 2:**
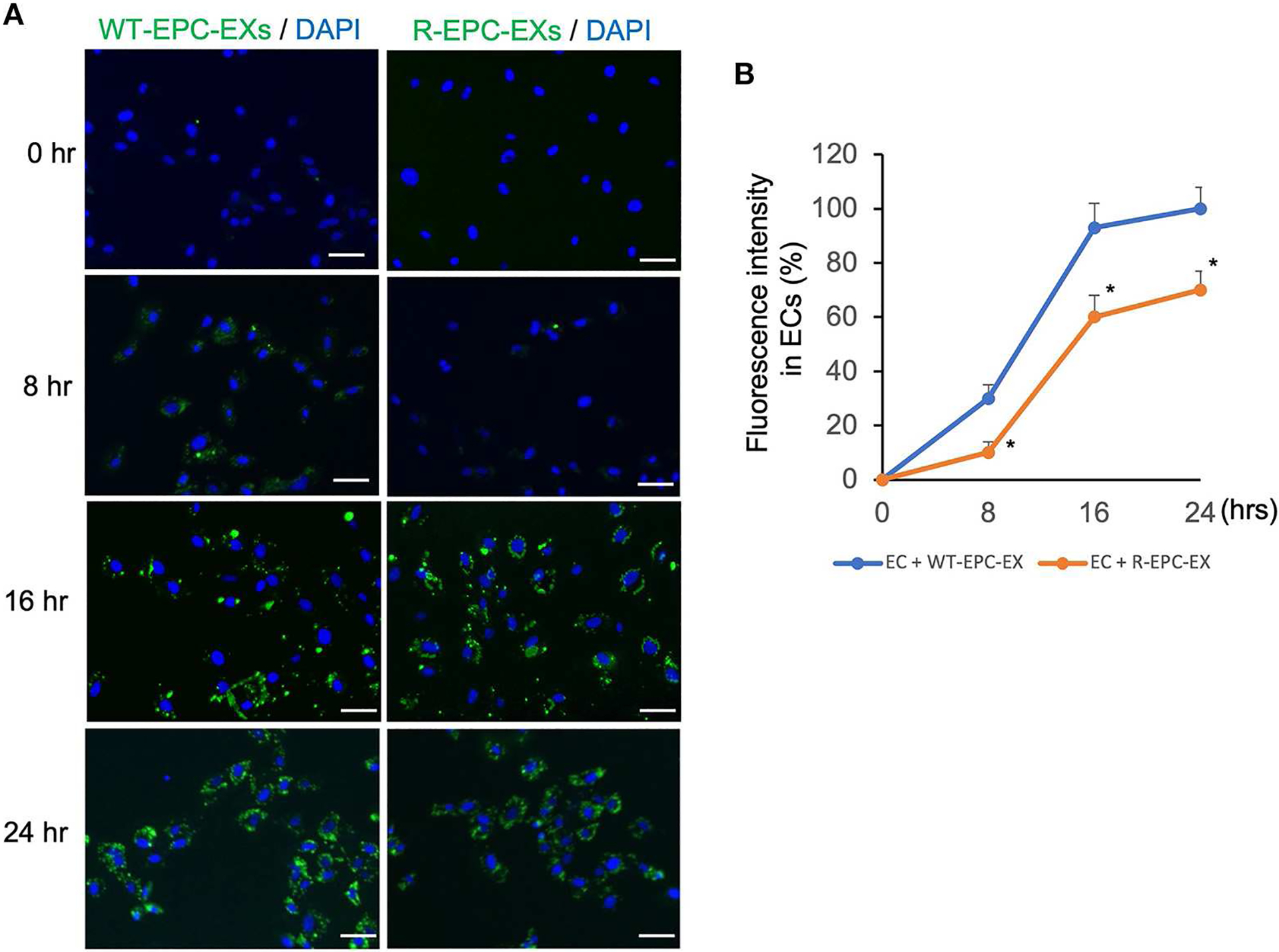
EPC-EXs derived from R+ mice have low incorporation rates into ECs. **(A)** representative images showing the incorporation of EX (labeled with PKH67, a green fluorescence) uptake by ECs at different incubation time points. Blue: DAPI. Scale bar: 50 um. **(B)** summarized data showing the time-dependent effect of EPC-EX incorporation into ECs. *p < 0.05, vs. WT-EPC-EX at the same time point. Data expressed as mean ± SEM, *n* = 6/group.

To analyze the uptake efficiency, EXs were labeled with PKH67 (green fluorescence) before being co-cultured with ECs. The uptake efficiency was determined by the fluorescence intensity in ECs at various co-incubation periods, 0, 8, 16, and 24 h. As shown in Figure 2, for the WT-EPC-EXs, the uptake efficiency was increased in a time-dependent pattern, as evidenced by a higher fluorescence intensity observed in the cytoplasm of target cells. It reached maximum internalization after 24 h co-incubation. For the ET-EPC-EXs, its internalization efficiency also occurred in a time-dependentmanner. Compared to the WT-EPC-EXs, the uptake efficiency of R-EPC-EXs by ECs (indicated by fluorescence intensity) was significantly lower at the same time. Together these data suggest that EPC-EXs mediated communication of EPC/EC is compromised in hypertensive R+ mice.

### The anti-apoptosis ability of EPC-EXs from hypertensive R+ mice on ECs is impaired

**FIGURE 3:**
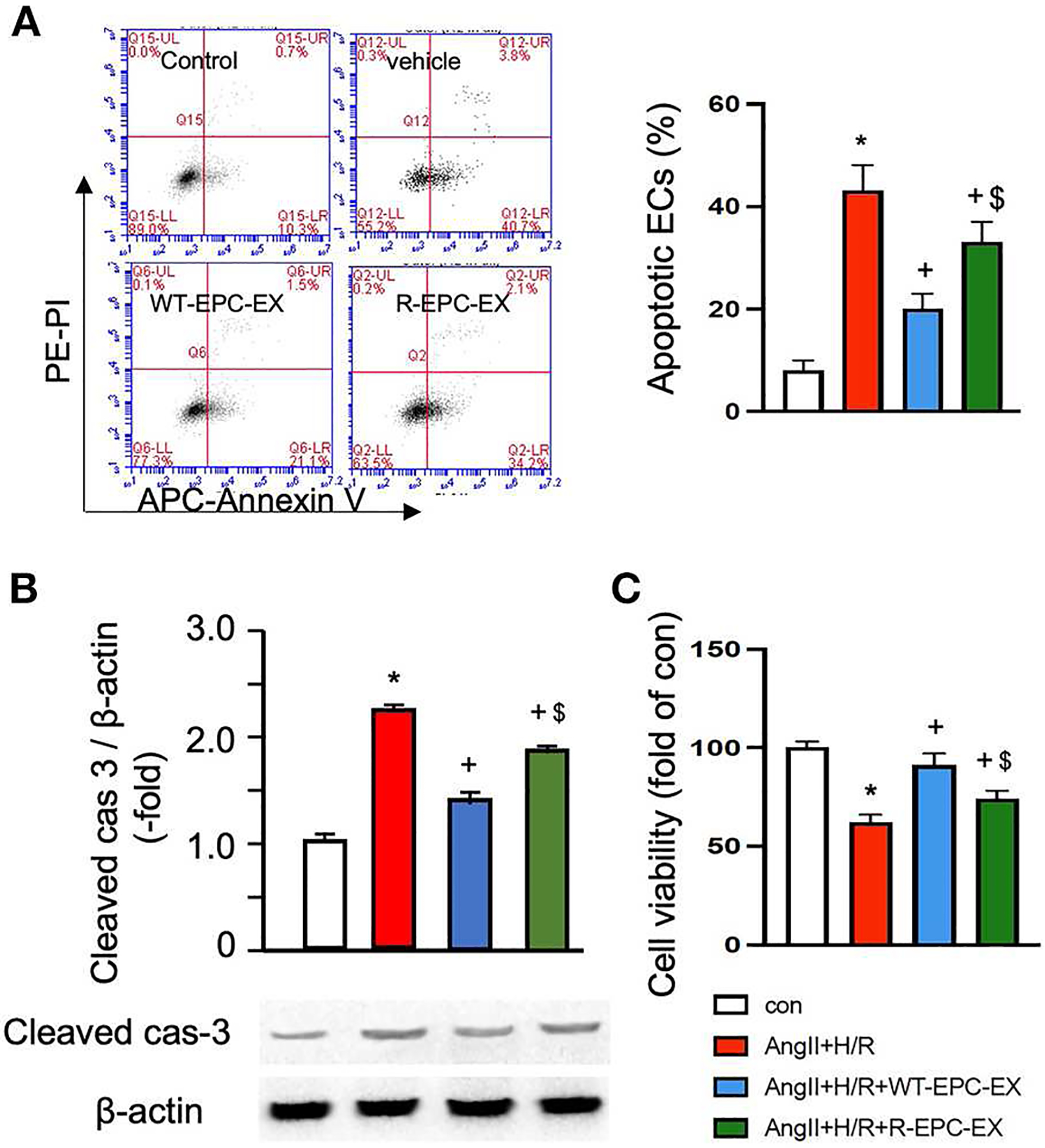
EPC-EXs derived from R+ mice are less effective on rescuing ECs from Ang II plus H/R-induced apoptosis and compromised cell viability. **(A)** representative plots, and summarized data of cell apoptosis. **(B)** cleaved cas-3/β-actin expression in ECs co-cultured with the two types of EPC-EXs. **(C)** cell viability of ECs co-cultured with the two types of EPC-EXs. *p < 0.05, vs. control; +*p* < 0.05, vs. vehicle; $*p* < 0.05, vs. WT-EPC-EXs. Data expressed as mean ± SEM, *n* = 6/group. EC+AngII+H/R: Mouse brain microvascular ECs (Cell Biologics) subjected to Ang II (10–6 M) plus hypoxia/reoxygenation (H/R) injury. Primary EC+H/R: primary brain microvascular ECs from R+ mice subjected to H/R injury. Control cells were cultured in normal conditions.

The EC injury model was induced by Ang II (10^−6^ M) combined with hypoxia/reoxygenation (H/R) to mimic hypertension-associated ischemic stroke injury. Cells were treated with vehicle (culture medium only) during reoxygenation, WT-EPC-EXs, or R-EPC-EXs (4 × 10^7^ EXs/ml) (Valadi et al., 2007). Our data (Figure 3A) showed that Ang II plus H/R challenge significantly increased the EC apoptosis, as evidenced by a higher percentage of cells expressing Annexin V (*p* < 0.05, vs. control). In the treatment groups, we found a relatively higher rate of Annexin V positive ECs in those co-incubated with R-EPC-EXs compared to that co-incubated with WT-EPC-EXs (*p* < 0.05, vs. Ang II + H/R+WT-EPC-EX). Meanwhile, we analyzed the caspase-3 expression in ECs after co-incubation. Our data (Figure 3B) showed that Ang II plus H/R challenge significantly raised the level of cleaved caspase-3 (*p* < 0.05, vs. con), which could be remarkably reduced by WT-EPC-EXs treatment (*p* < 0.05, vs. Ang II + H/R). As compared to WT-EPC-EXs, R-EPC-EXs elicited less effect in downregulating the cleaved caspase-3 in ECs (*p* < 0.05, vs. Ang II + H/R+WT-EPC-EXs). Similar effects were observed in cell viability as revealed by MTT assay. R-EPC-EXs had less efficiency than WT-EPC-EXs in rescuing ECs against Ang II plus H/R injury (Figure 3C).

Taken together, these data indicate that R-EPC-EXs had weaker anti-apoptotic ability than WT-EPC-EX in protecting ECs against hypertension-ischemia injury, reflecting that EPC-EX function is impaired in R+ mice, which might contribute to cerebral damage.

### The pro-angiogenic ability of EPC-EXs from hypertensive R+ mice on ECs is compromised

**FIGURE 4:**
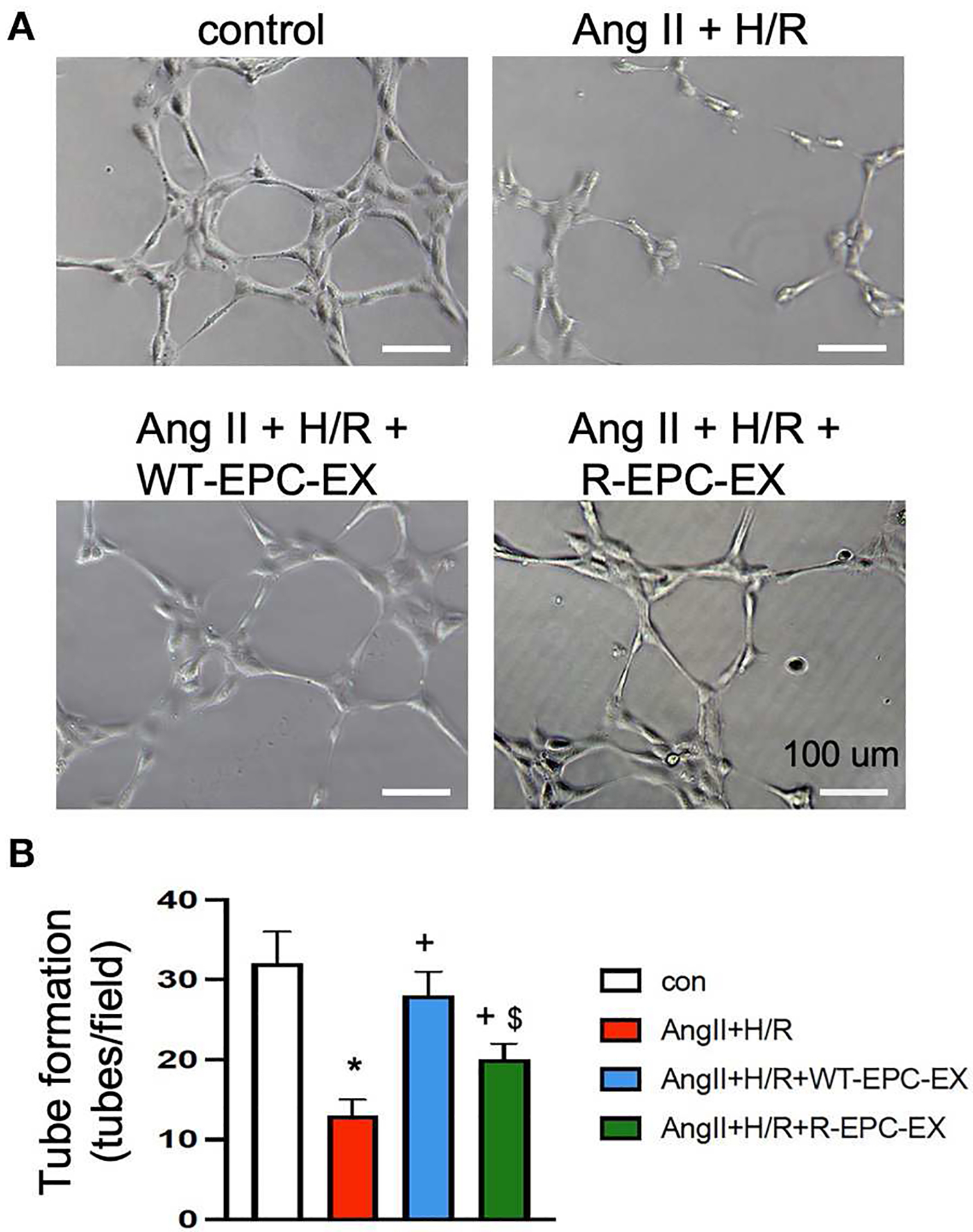
EPC-EXs derived from R+ mice are less effective in improving the tube formation ability of Ang II plus H/R-injured ECs. **(A,B)** representative images and summarized data showing the tube formation ability of ECs. *p < 0.05, vs. control; ^+^*p* < 0.05, vs. vehicle; ^$^*p* < 0.05, vs. WT-EPC-EXs. Data expressed as mean ± SEM, *n* = 6/group. EC+AngII+H/R: Mouse brain microvascular ECs (Cell Biologics) subjected to Ang II (10^−6^ M) plus hypoxia/reoxygenation (H/R) injury. Primary EC+H/R: primary brain microvascular ECs from R+ mice subjected to H/R injury. Control cells were cultured in normal conditions.

As revealed by *in vitro* tube formation assay (Figure 4), the Ang II plus H/R challenged ECs had a compromised ability of angiogenesis as indicated by fewer tubes formed in a field (*p* < 0.05, vs. control). To determine whether there is a difference in the pro-angiogenic ability of the two types of EPC-EXs, we treated the Ang II plus H/R injured ECs with EXs. Our data showed that WT-EPC-EXs remarkably improved the tube formation capacity of ECs (*p* < 0.05, vs. Ang II + H/R). In contrast, R-EPC-EXs only partially improved the pro-angiogenic ability of Ang II plus H/R challenged ECs (*p* < 0.05, vs. Ang II + H/R+WT-EPC-EX).

## Discussion

In the present study, our data have demonstrated that EPC-EXs from human renin transgene hypertensive (R+) mice exhibited reduced efficiency in rescuing brain microvascular ECs against Ang II plus hypoxia injury, suggesting EXs mediated intercellular communication between EPCs and brain ECs is compromised in hypertension conditions. Such impairment of the exosomal communication might contribute to the exaggerated cerebral ischemia injury in hypertension-associated ischemic stroke.

Emerging evidence indicates that EXs convey bioactive molecules to mediate intercellular communication (Valadi et al., 2007; Zhou et al., 2018; Pegtel and Gould, 2019; Zhang et al., 2019) and play critical roles in tissue “microenvironment” (Hofer and Tuan, 2016) that is involved in physiology and the development/progression of vascular diseases (Xin et al., 2014; Zhang and Chopp, 2016). In hypertension conditions, various types of EXs and their carried cargoes have been shown to be implicated in the pathogenesis of vascular complications. Plasma-derived EXs from spontaneous hypertension rats have been shown to stimulate aorta remodeling in the artery of normotensive rats (Otani et al., 2018). Another study showed that the exosomal miR miR-17-5p which is linked to inflammation was increased in spontaneous hypertensive rats compared to normotensive controls (Liu et al., 2019).

EPC-extracellular vesicles have been shown to promote angiogenesis (Deregibus et al., 2007), vascular repair (Li et al., 2016) and hindlimb ischemia recovery (Ranghino et al., 2012), suggesting EPC-EX-mediated communication is involved in ischemia tissue repair. Given that EX function varies upon the parent cell source and status (Yanez-Mo et al., 2015; Doyle and Wang, 2019; Zhang et al., 2019), we postulate that the declined EPC function in hypertension could affect EPC-EX communications with ECs, which will accelerate ischemic injury in the brain. To test this hypothesis, we first determined the internalization ability of EPC-EXs isolated from human renin transgene hypertensive (R+) mice and control mice by one of the major target cells in the brain microvascular ECs. We collected and analyzed the size of the two types of EXs prior to conducting *in vitro* exosomal functional study. Our data revealed no significant difference in the size distribution, reflecting that the size of EXs doesn’t significantly change in hypertension conditions. As increasing studies show that after internalization into recipient cells, EXs could release their carried cargoes, thereby altering target cells’ functions (Zhang et al., 2019). Several mechanisms of uptake have been proposed for EXs. Previous evidence indicates that EXs can be internalized by fusion and/or endocytosis (Gonda et al., 2019). Montecalvo et al. reported that with EXs could deliver their contents through the fusion or hemi-fusion between dendritic cells (Montecalvo et al., 2012). Monocyte-derived microvesicles can deliver their contents by fusion to platelets. The activated platelets can fuse with the microvesicle membrane more rapidly than unstimulated ones (Del Conde et al., 2005). We have evidence (data not shown here) that the EPC-EXs are uptaken by ECs in a caveolae-dependent mechanism. Theoretically, the more EXs were uptaken, the more cargoes would be released into the recipient cells. In our co-culture experiment set, intriguingly, we found that the uptake efficiency of EPC-EXs from the hypertensive R+ mice by ECs was significantly reduced compared to those EPC-EXs from the control mice at the same time point of co-incubation. This finding will help us to understand the difference in functions between the two types of EXs. Meanwhile, whether the uptake mechanism is related to the altered uptaken efficiency is unclear and will be further studied.

To further determine if there is a compromised communication between EPCs and ECs mediated by EXs, we conducted an *in vitro* co-culture study of EPC-EXs and ECs. To mimic the hypertension ischemia injury *in vitro*, we challenged the brain microvascular ECs with Ang II combined with hypoxia before co-incubating with EXs. The cell injury model was confirmed by cell viability and angiogenic ability analyses as revealed by increased cellular apoptosis, decreased cell proliferation ability, and reduced tube formation capability. This is partially in line with previous studies showing that hypoxia causes dysfunction of ECs (Cao et al., 2019) and indicates the success of the cellular model. As shown by the co-incubation study, we found that EPC-EXs from the control mice (WT-EPC-EXs) significantly rescued ECs from Ang II plus hypoxia damage, partially supported by previous studies showing that EPC-EXs could protect ECs against hypoxia injury (Wang et al., 2013). However, our data showed that such protection effects elicited by EPC-EXs from the hypertensive R+ mice (R-EPC-EXs) in ECs were remarkably decreased as revealed by a high percentage of EC apoptosis, a low cell proliferation rate, and compromised tube formation ability of ECs. These findings directly reflect that the beneficial effects of EPC-EXs were compromised in hypertension mice. These data suggest that the communication between EPCs and ECs might be disturbed in hypertension, contributing to the enlarged cerebral ischemia injury in hypertension. Although this is the first investigation to evaluate the exosomal-mediated communication of EPCs and ECs in hypertension hypoxia conditions, there is some previous evidence that supports our findings. For instance, Wu et al., have previously demonstrated that EPC-derived extracellular vesicles from uncontrolled type 2 diabetic patients have compromised ability to protect EPCs from hyperglycemia injury (Wu et al., 2016). Meanwhile, we have initial data (data not shown) showing that the miRNA profiling of EPC-EXs is altered in hypertension conditions. Whether such miR alteration contributes to their function difference and what are the underlying mechanism mediated by exosomal miRs are unknown.

There are several limitations of the present study. First, we just investigated the EX-mediated communication of EPCs and ECs in hypertension ischemia conditions *in vitro* cell culture systems. The relative communication *in vivo* needs to be further explored at least in a hypertension animal model. Second, we didn’t gain deep insight into the potential mechanism that could be responsible for the observed results. Whether the exosomal cargoes such as microRNAs or proteins contribute to the altered communication between EPCs and ECs in hypertension hypoxia conditions needs to be profoundly determined in the future.

## Conclusions

Taken together, our data have demonstrated that the communication between EPCs and ECs mediated by EPC-EXs is compromised in hypertension conditions. These findings provide new insights into exploring the mechanism of the enlarged cerebral ischemia injury in hypertension-associated ischemic stroke.

## Data Availability

The raw data supporting the conclusions of this article will be made available by the authors, without undue reservation.
